# High Expression of *ANXA2* Pseudogene *ANXA2P2* Promotes an Aggressive Phenotype in Hepatocellular Carcinoma

**DOI:** 10.1155/2019/9267046

**Published:** 2019-02-10

**Authors:** Qiu-shuang Wang, Liang-Liang Shi, Fei Sun, Yi-fan Zhang, Ren-Wang Chen, Sheng-li Yang, Jian-li Hu

**Affiliations:** ^1^Cancer Center, Union Hospital, Tongji Medical College, Huazhong University of Science and Technology, Wuhan 430022, China; ^2^Department of Orthopedics, Liyuan Hospital, Tongji Medical College, Huazhong University of Science and Technology, Wuhan 430077, China; ^3^Department of Hepatobiliary Surgery, The Affiliated Hospital of Guizhou Medical University, Guiyang, Guizhou 550001, China

## Abstract

**Objective:**

Accumulating evidence suggests that pseudogenes play potential roles in the regulation of their cognate wild-type genes, oncogenes, and tumor suppressor genes. ANXA2P2 (annexin A2 pseudogene 2) is one of three pseudogenes of annexin A2 that have recently been shown to be aberrantly transcribed in hepatocellular carcinoma (HCC) cells. However, its clinical meaning and biological function in HCC have remained unclear. Therefore, the present study was aimed at exploring the prognostic value of a high expression of ANXA2P2 in HCC tissue and at identifying whether it can affect the efficacy of targeted drugs (sorafenib, regorafenib, and lenvatinib).

**Methods:**

We obtained ANXA2P2 mRNA expression levels from The Cancer Genome Atlas (TCGA) RNA sequence database. The expression levels of ANXA2P2 in 49 pairs of intratumoral and peritumoral liver tissues were examined by RT-PCR. Wound healing and transwell assays were performed to confirm the tumor-promoting properties of ANXA2P2 in HCC cells. CCK8 assay was conducted to identify whether ANXA2P2 can affect the growth of HCC cells when administered with targeted drugs (sorafenib, regorafenib, and lenvatinib).

**Results:**

The expression of ANXA2P2 in HCC tissues was significantly higher than that in adjacent cancerous tissues from TCGA database and validation group. Additionally, patients with high ANXA2P2 expression in HCC tissue had a shorter overall survival, whereas no statistically significant correlation was found between ANXA2P2 expression and disease-free survival (*p* = 0.08) as well as other clinical parameters, such as age, gender, histological grade, T classification, stage, albumin level, alpha-fetoprotein, and vascular invasion (*p* = 0.7323, 0.8807, 0.5762, 0.8515, 0.7113, 0.242, 1.0000, and 0.7685, respectively). Furthermore, *in vitro* experiments showed that knockdown of ANXA2P2 inhibited migration and invasion of HCC cells but did not have an influence on the HCC cell proliferation when treated with targeted drugs (sorafenib, regorafenib, and lenvatinib).

**Conclusion:**

Our study confirmed elevated ANXA2P2 expression levels in HCC tissue compared with adjacent noncancerous tissue and a worse prognosis of patients with high ANXA2P2 levels in the HCC tissue. The newly found properties of promoting migration and invasion of ANXA2P2 in HCC help to explain this phenomenon. ANXA2P2 could be a novel and suitable predicative biomarker for the risk assessment of recurrence or metastasis of HCC patients but may not be effective to predict the efficacy of targeted drugs.

## 1. Introduction

Hepatocellular carcinoma (HCC) ranks as the fifth and seventh most common cancer in men and women, respectively [1], and the third leading cause of cancer-related mortality worldwide [2]. This disease is characterized by a high recurrence rate after curative resection and resistance to chemotherapy. Moreover, most HCC patients are in advanced stage at the time of diagnosis due to the lack of precise early diagnosis and meaningful treatment [3], causing a poor prognosis for a great part of the patients. Thus, novel candidate biomarkers for diagnosis, prognosis, and treatment are urgently needed.

Annexin A2 is a discriminative serological candidate in early hepatocellular carcinoma [4]. Structurally, annexin A2 is a 36 kDa peripheral membrane protein that belongs to the annexin family of calcium- and phospholipid-binding proteins composed of 12 paralogs in humans and is originally characterized by the ability to bind and aggregate anionic phospholipid membranes in a calcium-dependent manner [5]. Abnormal expression of annexin A2 was found in hepatocellular carcinoma and other types of tumors, such as pancreatic cancer [6, 7], gastric adenocarcinoma [8], prostate carcinoma [9], high-grade glioma [10], oral squamous cell carcinoma [11], and breast cancer [12]. Overexpression of annexin 2 is involved in several pathological processes, such as tumor cell adhesion, proliferation, apoptosis, tumor neoangiogenesis, invasion, and metastasis [13]. High expression of annexin A2 is associated with a worse survival rate of HCC patients [14].

Pseudogenes are genomic loci that resemble their cognate genes and were classically thought of as nonfunctional DNA sequences due to their defects that either prevent transcription or result in the generation of nonfunctional proteins [15]. Increasing evidence strongly indicates that pseudogenes have diverse biological functions in pathological and physiological processes and especially in human cancer progression [16]. Pseudogenes can play potential roles in regulating cognate wild-type gene expression/function by serving as endogenous siRNA, antisense transcripts, competitive inhibitors of the translation of wild-type transcripts, and possibly dominant-negative peptides [17]. Recently, progress in microarray and bioinformatic technologies facilitated the detection of pseudogene transcription at the whole-genome level and has revealed that numerous pseudogenes are indeed transcribed [18]. In total, 2082 pseudogenes are ubiquitously expressed in human cells, and among them, 218 were found to be expressed only in cancer cells, with 40 being restricted to a single cancer type [16]. Furthermore, recent studies also suggest that pseudogenes are involved in the regulation of other oncogenes and tumor suppressor genes apart from their cognate wild-type genes [19].

It has been found that the functional annexin A2 gene maps to chromosome 15q22.2, whose three pseudogenes map to chromosomes 4q21-q31, 9p13, and 10q21–22 (ANXA2P1, ANXA2P2, and ANXA2P3, respectively) [5]. Although containing no deletions, they exhibit point mutations, which impede the production of proteins from the identified transcripts [5]. ANXA2P2 (annexin A2 pseudogene 2, also known as ANX2L2, ANX2P2, or LPC2B), a processed pseudogene that is highly homologous to annexin A2, has recently been shown to be transcribed in malignant diffuse glioma cells [20]. Overexpression of ANXA2 predicts adverse outcomes in patients with malignant tumors [13]. However, it is unknown whether its pseudogene ANXA2P2 has the same predictive value, and the clinical meaning and biological function of ANXA2P2 in HCC have also remained unclear. The present study was aimed at exploring the prognostic value of a high expression of ANXA2P2 in HCC tissue, and this study is, to the best of our knowledge, the first report illustrating the role of ANXA2P2 in the invasion of HCC cells.

Sorafenib is the only FDA-approved first-line systemic targeted drug for advanced hepatocellular carcinoma [21], but its effect on patient survival is limited, because of primary drug resistance or acquired drug resistance [22]. Recently, according to the data of the RESORCE trial, a placebo-controlled phase III study that estimated the efficacy and safety of regorafenib in patients with HCC after systemic first-line treatment with sorafenib, regorafenib treatment contributed to a 2.8-month survival extension compared to placebo (10.6 months vs. 7.8 months) [23]. New drugs—lenvatinib in the frontline—have also been demonstrated to improve clinical outcomes [23, 24]. Research on biomarkers of a response or primary resistance to targeted drug is also advancing. For example, a recent study found that ANXA3 confers HCC cells with the ability to resist sorafenib [25]. Anti-ANXA3 monoclonal antibody therapy combined with sorafenib/regorafenib reduced HCC cell proliferation *in vivo* and significantly improved survival [25]. Furthermore, coexpression of ANXA2 conserved the chemoresistant ability in NRBP2-upregulating HCC cells [26]. Nevertheless, whether pseudogene ANXA2P2 could affect the efficacy of targeted drugs (sorafenib, regorafenib, and lenvatinib) has not been reported yet. Therefore, our study will preliminarily study if there is any correlation between ANXA2P2 and the efficacy of targeted drugs.

## 2. Methods

### 2.1. Patients and Their Clinicopathological Data from TCGA Database

We obtained ANXA2P2 mRNA expression levels from The Cancer Genome Atlas (TCGA) RNA sequence database (https://genome-cancer.ucsc.edu/). Patients meeting the following inclusion criteria were included in this study: pathological diagnosis with HCC, absence of pretreatment, and complete disease-free survival (DFS), overall survival (OS), and clinicopathological information.

### 2.2. Clinical Samples

In total, 49 pairs of archived, formalin-fixed, and paraffin-embedded intratumoral and peritumoral liver tissue specimens were obtained from 49 patients with pathologically proven HCC who underwent curative resection between November 1995 and May 2017 at the Prince of Wales Hospital (Hong Kong, China). Informed written consent was obtained from all patients involved in this study. The study was approved by the Joint Chinese University of Hong Kong-New Territories East Cluster Clinical Research Ethics Committee (Hong Kong, China). All methods were performed in accordance with the relevant guidelines and regulations.

### 2.3. HCC Cell Lines and Cell Culture

The human HCC cell line Hep3B was originally obtained from the China Center for Type Culture Collection (CCTCC, Wuhan, China). The cell lines were cultured in RPMI 1640 medium (Life Technologies, Beijing, China) and supplemented with 10% fetal bovine serum (FBS; Life Technologies, Beijing, China). The cells were maintained at 37°C in a humidified atmosphere containing 5% CO_2_.

### 2.4. Real-Time PCR

Total RNA was extracted from tissue and Hep3B cells using TRIzol (Invitrogen), and cDNA was synthesized using the RevertAid First-Strand cDNA Synthesis Kit (Thermo, code no. #K1622). Real-time PCR was performed using FastStart Universal SYBR Green Master (Rox) (Roche, code no. 04913 914001) on the LightCycler® 480 system (Roche Diagnostics) according to the manufacturer's protocol. The qRT-PCR reaction included an initial denaturation step at 95°C for 10 min, followed by 40 cycles at 95°C for 15 s and 60°C for 60 s, and at 75°C to 95°C; the temperature rises at 1°C per 20 s. The sequences for sense (S) and antisense (A) primers were as follows: human-ANXA2P2-S—5′-GTTAAAGGAGACCTGGAAAATGCT-3′—and human-ANXA2P2-A—5′-GGTAGTCGCCCTTAGTGTCTTG-3′.

### 2.5. Transfection

si-ANXA2P2 and its negative control siRNA (NC siRNA) were purchased from GenePharma (Shanghai, China) and transfected into Hep3B cells using Lipofectamine 2000 reagent (Invitrogen). Hep3B cells were seeded into six-well plates with 2 × 10^5^ cells per well and incubated for 24 h.

### 2.6. Transwell Assay

The invasion abilities of Hep3B cells were assessed using Transwell cell culture inserts (Costar Corporation). 2 × 10^4^ Hep3B cells transfected with no treatment, empty vector, or si-ANXA2P2 were plated into each Transwell cell culture insert in serum-free DMEM, whereas the lower chambers were plated with 700 *μ*L DMEM containing 10% FBS. After incubation for 48 h, the cells on the surfaces in the upper chambers were removed with cotton swabs, and the inner migrated cells were fixed with 500 *μ*L paraformaldehyde (4%) for 15 min. The membrane was washed with PBS and stained with crystal violet solution for 20 min. The migrated cells were counted and photographed with a microscope (Olympus, Japan).

### 2.7. Wound Healing Assay

The cell migration ability was measured using the scratch wound healing assay. After the Hep3B cells transfected with no treatment, empty vector, or si-ANXA2P2 was grown in a six-well plate to 90% confluence, and a micropipette tip was used to scratch a wound. The cellular debris was washed three times with serum-free medium, and the wound area was photographed under an Upright Metallurgical microscope (Olympus, Japan) at 0, 24, and 48 h.

### 2.8. Cell Proliferation Assay

For Cell Counting Kit-8 (CCK8; Corning, Japan) assays, Hep3B cells were transfected with no treatment, empty vector, or si-ANXA2P2; the transfected cells were plated in 96-well plates at a concentration of 2.5 × 10^3^ cells/well and maintained in 10% FBS for 24 h. Next, lenvatinib (4 *μ*mol/L and 8 *μ*mol/L), sorafenib (5 *μ*mol/L and 10 *μ*mol/L), and regorafenib (10 *μ*mol/L and 2 *μ*mol/L) with 200 *μ*L per well were, respectively, added. After 24 h, 48 h, and 72 h, the transfected Hep3B cells were replaced with 10% CCK8 diluted with normal culture medium and incubated for another 2 h. The absorbance of each well was measured with a microplate reader at 450 nm. All experiments were performed in triplicate.

### 2.9. Statistical Analysis

Statistical analyses were performed with the SPSS 19.0 software (IBM SPSS, Chicago, IL, USA). Survival analysis was performed by the Kaplan–Meier method with the log-rank (Mantel-Haenszel) test. 384 patients were involved in Kaplan–Meier survival analysis. After excluding incomplete clinical data obtained from TCGA dataset, 207 cases (Table 1) were analyzed by the chi-square test. GraphPad Prism version 5.0 (GraphPad Software, Inc., La Jolla, CA, USA) was used for graphing and analysis. Data are expressed as the means ± standard deviation (SD). The statistical significance of differences between two groups was assessed using two-sided Student's *t*-test, and values of *p* < 0.05 were considered statistically significant.

## 3. Results

### 3.1. Intratumoral and Peritumoral Expression Levels of ANXA2P2 in HCC from TCGA Database and Validation Group

**Figure 1:**
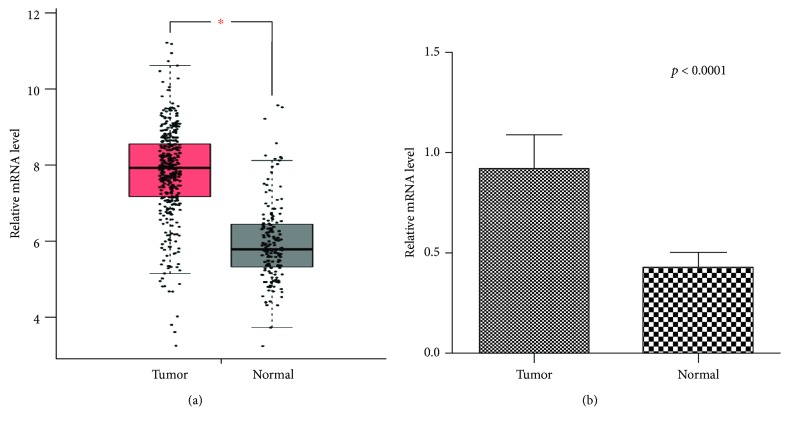
qRT-PCR analysis of ANXA2P2 expression levels in tumor and nontumor tissues of hepatocellular carcinoma patients. (a) 369 cases of hepatocellular carcinoma (HCC) tissues and 160 cases of adjacent nontumor tissues from TCGA dataset. (b) 49 pairs of tumor and nontumor tissues of HCC patients in the validation group. The ANXA2P2 expression level in tumor tissues is remarkably higher than that in nontumor tissues of HCC patients (*p* < 0.0001).

We found that the expression level of ANXA2P2 was remarkably higher in HCC tissues than in paracancerous tissues from TCGA database (Figure 1(a)). The ANXA2P2 expression levels in cancer tissues and adjacent nontumor tissues in the validation group, consisting of 49 patients, were detected and verified by qRT-PCR (Figure 1(b)). The results of the validation group were consistent with the results obtained from TCGA database.

### 3.2. High ANXA2P2 Expression Correlates with Worse Survival Outcome in TCGA Database

**Figure 2:**
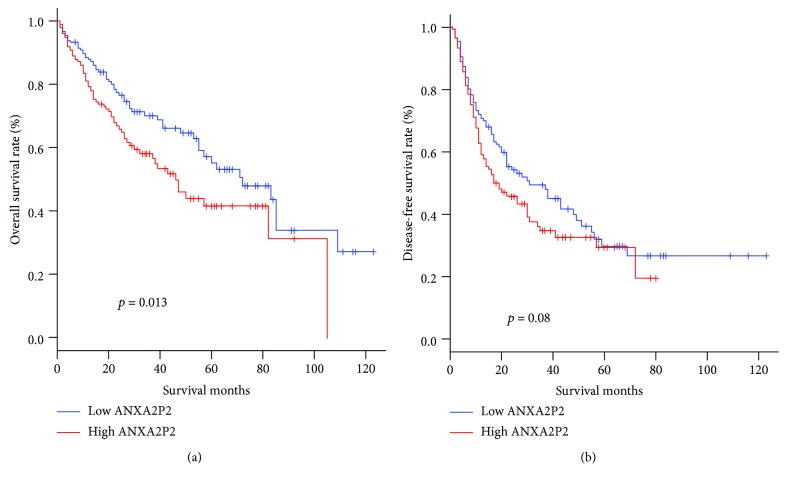
High expression levels of ANXA2P2 are associated with poor overall survival of hepatocellular carcinoma patients. Kaplan–Meier survival curve analysis with log-rank comparison was performed according to the ANXA2P2 expression level in TCGA dataset (364 cases, among them 182 cases with high and 182 with low expression levels). (a) The amplification of ANXA2P2 is associated with the OS of hepatocellular carcinoma (HCC) patients in TCGA dataset (*p* = 0.013). (b) ANXA2P2 expression level is not statistically associated with the DFS of HCC patients in TCGA dataset (*p* = 0.08).

Kaplan–Meier survival analysis showed that the OS of HCC patients with high ANXA2P2 levels was shorter than that of patients with low ANXA2P2 levels (Figure 2(a)), but there was no significant correlation between ANXA2P2 expression level and DFS (Figure 2(b)).

The chi-square test was performed to analyze the correlation between ANXA2P2 expression level and clinical parameters extracted from TCGA database. The results showed that there is no statistically significant correlation between ANXA2P2 expression and DFS (*p* = 0.08) as well as other clinical parameters, such as age, gender, histological grade, T classification, stage, albumin, AFP, and vascular invasion (*p* = 0.7323, 0.8807, 0.5762, 0.8515, 0.7113, 0.242, 1.0000, and 0.7685, respectively) (Table 2).

### 3.3. In Vitro Properties of ANXA2P2 That Promote Migration and Invasion in HCC

**Figure 3:**
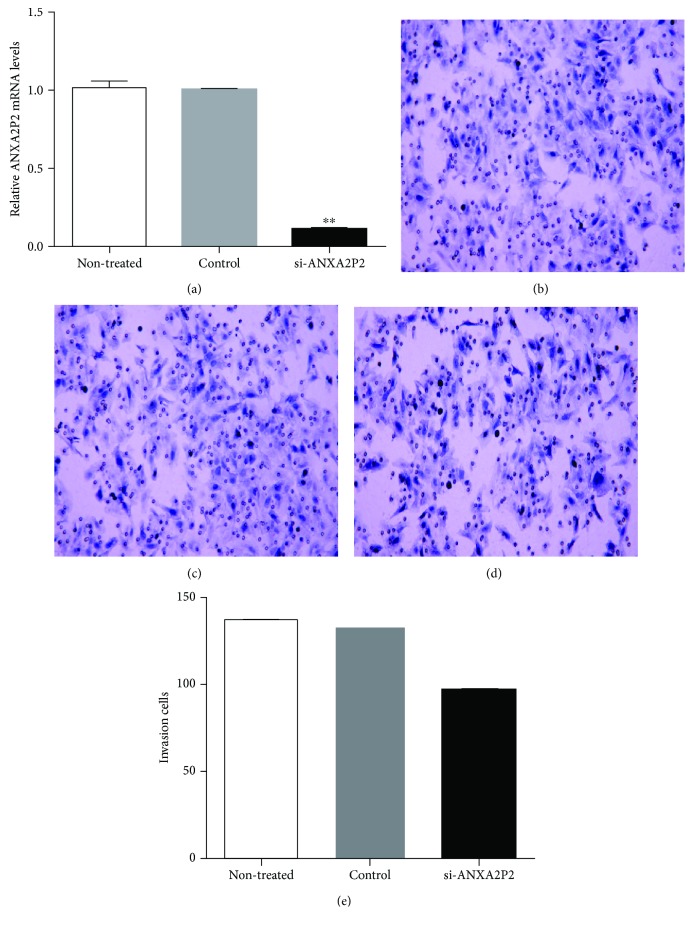
Transwell assay of Hep3B cells transfected with si-ANXA2P2 or negative control siRNA. (a) The mRNA expression levels of ANXA2P2 of Hep3B cells analyzed by real-time PCR following transfection with no treatment, negative control siRNA, and si-ANXA2P2. (b) Transwell assay of Hep3B cells transfected with no treatment. (c) Transwell assay of Hep3B cells transfected with negative control siRNA. (d) Transwell assay of Hep3B cells transfected with si-ANXA2P2. (e) Quantification of the invasion cells in the no-treatment group, negative control siRNA group, and si-ANXA2P2 group was shown.

**Figure 4:**
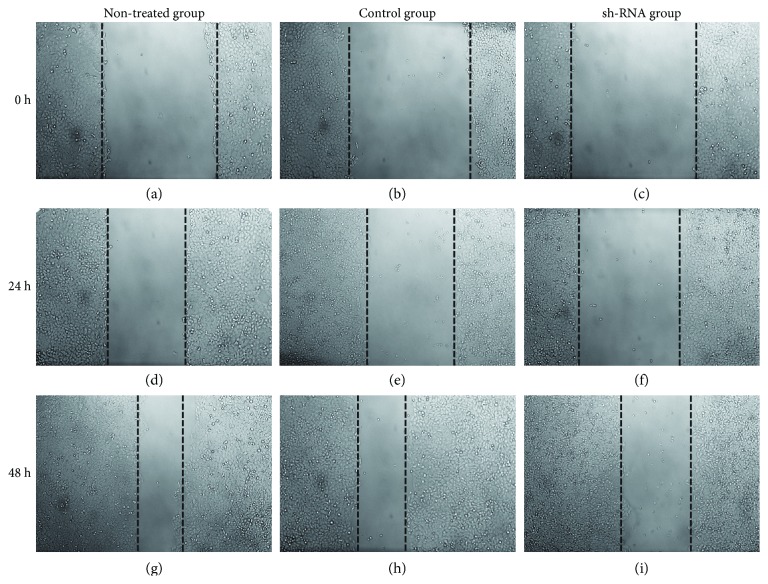
Wound healing assay of Hep3B cells transfected with no treatment, negative control siRNA, or si-ANXA2P2 photographed at 0, 24, and 48 h. Hep3B cells transfected with si-ANXA2P2 exhibited distinctly decreased migration and invasion ability after 0 (a, b, c), 24 (d, e, f), and 48 h (g, h, i).

The mRNA expression levels of ANXA2P2 in the si-ANXA2P2 group were obviously declined compared to the no-treatment group and negative control siRNA group (Figure 3(a)). The transwell assay showed that the density of hepatoma cells in the si-ANXA2P2 group was significantly lower than that in the control group (Figures 3(b)–3(d)), and the number of the invasion cells was remarkably decreased in the si-ANXA2P2 group (Figure 3(e)). Accordingly, we concluded that ANXA2P2 promotes the migration and invasion of HCC cells. Furthermore, the cell scratch test showed that the hematoma cells of the si-ANXA2P2 group barely crossed the midline (Figure 4). However, the hematoma cells in the negative control siRNA group (NC siRNA) obviously crossed the midline over time (photographed at 0, 24, and 48 h, respectively) (Figure 4).

### 3.4. In Vitro ANXA2P2 Has No Impact on the Proliferation of Hep3B Cells or the Efficacy of Targeted Drugs (Sorafenib, Regorafenib, and Lenvatinib) in HCC

**Figure 5:**
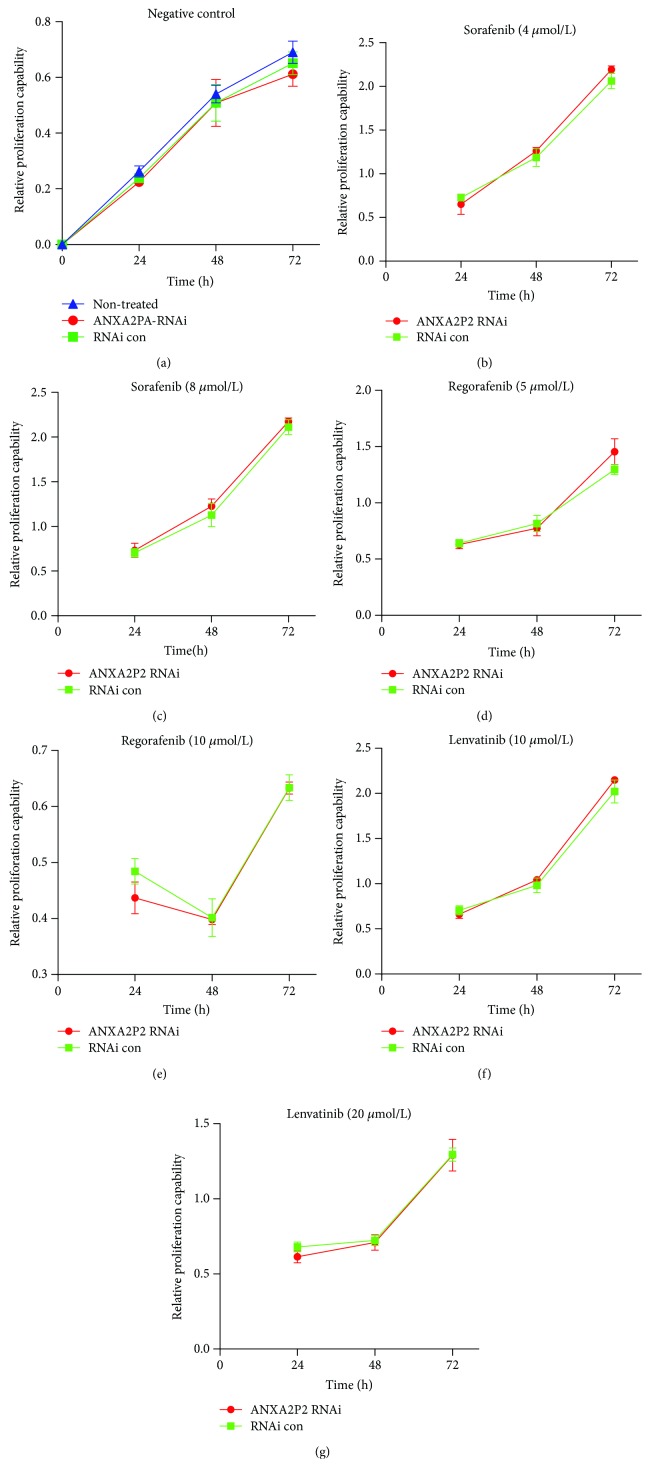
CCK8 assay of Hep3B cells transfected with si-ANXA2P2 or negative control siRNA when treated with negative control, sorafenib (4 *μ*mol/L, 8 *μ*mol/L), regorafenib (5 *μ*mol/L, 10 *μ*mol/L), and lenvatinib (10 *μ*mol/L, 20 *μ*mol/L), respectively, for three days and measured at the time points of 24, 48, and 72 h. There was no significant difference in relative proliferation capability between the no-treatment group, negative control siRNA group, and si-ANXA2P2 group (a). The relative proliferation capability was not significantly different between the negative control siRNA group and the si-ANXA2P2 group when administered with sorafenib (b, c), regorafenib (d, e), and lenvatinib (f, g) with different concentrations at the time points of 24, 48, and 72 h.

To determine the effect of ANXA2P2 on the growth of HCC cells, we used transient transfection to downregulate its expression. The results showed no significant difference in the proliferation of HCC cells between the no-treatment group, the negative control siRNA group (NC siRNA), and the si-ANXA2P2 group (Figure 5(a)). To further investigate whether ANXA2P2 can affect the efficacy of current liver cancer-targeted drugs, the si-ANXA2P2 group and the negative control were treated with sorafenib (4 *μ*mol/L, 8 *μ*mol/L), regorafenib (5 *μ*mol/L, 10 *μ*mol/L), and lenvatinib (10 *μ*mol/L, 20 *μ*mol/L), respectively, for three days, as described in Methods. The results showed that there was no significant difference in relative proliferation capability between the si-ANXA2P2 group and the negative control siRNA when exposed to sorafenib (Figures 5(b) and 5(c)), regorafenib (Figures 5(d) and 5(e)), and lenvatinib (Figures 5(f) and 5(g)) at different concentrations at the time points of 24, 48, and 72 h.

## 4. Discussion

In this study, we first identified and verified the role of the ANXA2 pseudogene ANXA2P2 in HCC. Results obtained from TCGA database and the validation group of 49 HCC patients were in good agreement and showed that the expression of ANXA2P2 was significantly upregulated in HCC tissue. Furthermore, Kaplan–Meier curves analyzed from TCGA database showed that high ANXA2P2 expression levels were correlated with a poor OS of HCC patients. Subsequently, results of transwell assay and wound healing assay further confirmed the tumor-promoting properties of ANXA2P2 in HCC cells, and CCK8 assay demonstrated that ANXA2P2 has no impact on the proliferation of HCC cells, or when treated with targeted drugs, suggesting that ANXA2P2 may be a suitable predicative biomarker for risk assessment of recurrence or metastasis of HCC patients.

In HCC, the expression of ANXA2 was higher in tumor tissue than in nontumor tissue [27]. Furthermore, ANXA2 expression was significantly correlated with differentiated degree, intrahepatic metastasis, portal vein thrombus, and tumor node metastasis (TNM) staging [27]. More importantly, overexpression of ANXA2 was closely associated with a shortened overall survival (OS) of HCC and identified as an independent prognostic factor [4, 27, 28]. In addition, downregulation of ANXA2 impaired cell proliferation and motility, enhanced apoptosis, and inhibited cell pseudopodia/filopodia by suppressing the expression of *β*-tubulin and F-actin [14]. Several studies have confirmed that serum ANXA2 may serve as a biomarker for the early detection of HCC [4, 27]. Therefore, in the present study, we mainly focused on the pseudogene of ANXA2 (ANXA2P2).

In general, pseudogenes are classified into three categories: processed (or retrotransposed), unprocessed (or duplicated), and unitary pseudogenes [18]. Processed pseudogenes are the most abundant type in the human genome. They originate from retrotransposition, the transcription of a parental gene into normal mRNA, which is reversely transcribed into cDNA [18]. This newly formed cDNA is retrotransposed at another position in the genome. Therefore, processed pseudogenes are intronless and have a poly A tail at the 3′ end flanking direct repeats. The formation of the double-stranded structures of processed pseudogenes from single-stranded RNA is catalyzed by RNA polymerase III [18, 19, 29]. Unprocessed pseudogenes may result from various modifications, including point mutations, frameshifting events, insertions, and deletions [18, 19].

ANXA2P2 is one of three pseudogenes mapping to chromosomes 4q21-q31, 9p13, and 10q21-22 (ANXA2P1, ANXA2P2, and ANXA2P3, respectively) and presents a kind of processed pseudogene [5]. A recent report revealed that the expression of ANXA2 is positively correlated with the expression of ANXA2P2 [20]. Furthermore, high expression of the pseudogenes ANXA2 and annexin A2 is associated with the poor survival outcome in diffuse glioma patients [20]. However, reports about the pseudogenes of ANXA2 are very limited, and no study focuses solely on ANXA2P2 in HCC. The present report presents, to the best of our knowledge, the first study to identify the expression pattern and underlying predictive value of ANXA2P2 in HCC, indicating that pseudogenes of ANXA2 may play important roles in HCC progression or metastasis.

Pseudogenes have been considered nonfunctional “genomic fossil” or “junk genes.” However, emerging studies revealed that some pseudogenes play crucial roles in human cancer [19]. For instance, high expression levels of POU5F1P4 (a pseudogene of OCT4) were found to be statistically significantly correlated with a worse prognosis of HCC patients [30]. Furthermore, high expression of PTENP1 (a pseudogene of PTEN) might result in better OS and DFS rates of head and neck squamous cell carcinoma (HNSCC) patients [31]. SFTA1P is pseudogene-derived long noncoding RNA and strongly associated with advanced tumor/lymph node/metastasis (TNM) stage, lymphatic metastasis, larger tumor volume, and worse prognosis of gastric cancer (GC) patients [32]. DUXAP8 is a transcribed pseudogene that is upregulated in non-small-cell lung cancer (NSCLC) tissues, and higher DUXAP8 expression is correlated with shorter overall survival in NSCLC [33]. In the present study, we found that high expression of ANXA2 pseudogene 2 is correlated with the poor OS of HCC patients. According to which mechanism do pseudogenes probably affect cancer cell progression? The pseudogene POU5F1P4 acts as a competitive endogenous RNA (ceRNA) to prevent POU5F1 transcription from being inhibited by miR-145, thus promoting HCC cell growth and tumorigenicity [30]. The pseudogene PTENP1 plays a role in inhibiting tumor growth by negatively regulating the expression of PTEN [34]. Due to the high-homology region within its gene sequence and the resulting highly homologous properties, PTENP1 competitively inhibited the transcription of PTEN-related miRNAs, such as miR-17, miR-19b, and miR-20a [31, 34]. The transcript of the HMGA1 pseudogene HMGA1-p acts as a decoy for the HMGA1-targeting miRNAs; in addition, elevated levels of HMGA1-p RNA could destabilize and thus accelerate the degradation of the HMGA1 mRNA transcript to display its oncogenic properties [35]. Furthermore, it has been reported that the transcript of the pseudogene *ψ*PPM1K could develop a tumor-suppressing ability independent of its parental gene [36]. In the present study, we found that the expression level of ANXA2P2 was remarkably increased in HCC tissue, which is correlated with the poor OS of HCC patients. In light of previously described pseudogenes and their wild-type parental genes and because of the high homology of the gene sequence and significant clinical correlation, we scientifically and reasonably deduced that ANXA2P2 transcript and ANXA2 mRNA can interact directly or indirectly to promote tumor cell invasion or metastasis. This mechanism is similar to the functional mechanism of the pseudogenes mentioned above.

Interestingly, CCK8 assay showed that ANXA2P2 did not affect the proliferation of HCC cells compared with transwell assay and wound healing assay, suggesting that ANXA2P2 could better predict invasion and metastasis, rather than proliferation. A recent study revealed that ANXA2 enhances the progression of the HCC cell via remodeling the cell movement [14], which to some extent supports the result of our experiment and hypothesis that ANXA2 mRNA and ANXA2P2 may interact directly or indirectly to promote tumor cell invasion or metastasis through remodeling the HCC cell movement. Although there are very few reports on direct upstream and downstream targets of ANXA2P2, we speculated that a further mechanistic study is worthy of extended experimental and clinical investigation. Simultaneously, when sorafenib, regorafenib, and lenvatinib were administrated at different concentrations, results showed that there is no significant difference in relative proliferation capability between the si-ANXA2P2 group and the negative control siRNA group (Figures 5(b)–5(g)), indicating that ANXA2P2 may not mainly participate in targeted drug-related signaling pathways or may not be located at the common hub of the signaling pathway. It has been manifested that ANXA3 conferred ability to resist sorafenib upon HCC cells by suppressing PKC*δ*/p38-related apoptosis and activated autophagy for cell survival [25]. Clinically, upregulation of ANXA3 was associated with a shorter overall survival time in HCC patients who continue to accept sorafenib treatment [25]. ANXA2 and ANXA3, as two different annexin family members, rarely interfere or interact with each other [37]; therefore, it is possible that ANXA2P2 would not have an influence on the efficacy of targeted drugs (sorafenib, regorafenib, and lenvatinib).

Our study exhibits two limitations. Firstly, since a large body of studies has reported the expression of ANXA2 in HCC and its relationship with the prognosis of HCC patients, we did not explore ANXA2 in the present study. In addition, we infer the interaction between ANXA2 mRNA and ANXA2P2 transcript from reported as well as our current data, and we will verify this conjecture experimentally in the future.

In conclusion, our study confirmed the relationship between ANXA2P2 and the prognosis of HCC patients by showing that ANXA2P2 expression levels of HCC tissues are higher than those of adjacent nontumor tissues and that the prognosis of patients with high ANXA2P2 expression levels in HCC tissue is worse. One of the possible mechanisms explaining the relationship between ANXA2P2 and HCC presents the promotion of HCC cell migration and invasion by ANXA2P2, expanding the scope of this mechanism involved in the development of HCC.

## Figures and Tables

**Table 1:** Main demographic, biochemical, and clinical characteristics of the 207 HCC patients.

Variable	Unit	Value
Age	Years	60 (16–85)
Gender	Male	143 (69.5%)
Albumin	g/L	40 (2-41)
AFP	ng/mL	13 (1-308836)

Data are presented as the median value (range) or absolute frequency (%) of alanine aminotransferase; HCC: hepatocellular carcinoma.

**Table 2:** Correlations of ANXA2P2 amplification in HCC tissues with clinicopathological characteristics from TCGA database.

Parameter	ANXA2P2		*p* value
	High	Low	
Age			0.7323
≥50 y	84	81	
<50 y	20	22	
Gender			0.8807
Male	71	72	
Female	33	31	
Histological grade			0.5762
G1-2	56	60	
G3-4	48	43	
T classification			0.8515
T1-2	88	86	
T3-4	16	17	
Stage			0.7113
I-II	88	85	
III-IV	16	18	
Albumin			0.242
≤35 g/L	27	19	
>35 g/L	77	84	
AFP			1.0000
<400 *μ*g/L	79	79	
≥400 *μ*g/L	25	24	
Vascular invasion			0.7685
Presence	36	33	
Absence	68	70	

## Data Availability

All data generated or analyzed during this study are included in this published article.

## References

[1] Chen W., Zheng R., Baade P. D. (2016). Cancer statistics in China, 2015. *CA: A Cancer Journal for Clinicians*.

[2] Njei B., Rotman Y., Ditah I., Lim J. K. (2015). Emerging trends in hepatocellular carcinoma incidence and mortality. *Hepatology*.

[3] Utsunomiya T., Shimada M., Imura S., Morine Y., Ikemoto T., Mori M. (2010). Molecular signatures of noncancerous liver tissue can predict the risk for late recurrence of hepatocellular carcinoma. *Journal of Gastroenterology*.

[4] Sun Y., Gao G., Cai J. (2013). Annexin A2 is a discriminative serological candidate in early hepatocellular carcinoma. *Carcinogenesis*.

[5] Ozeki M., Hoshino S., Hiai H., Toyokuni S. (2002). Isolation and characterization of *annexin* 2 pseudogene in *Rattus norvegicus*. *Gene*.

[6] Sato T., Kita K., Sugaya S., Suzuki T., Suzuki N. (2012). Extracellular release of annexin II from pancreatic cancer cells and resistance to anticancer drug-induced apoptosis by supplementation of recombinant annexin II. *Pancreas*.

[7] Huang Y. K., Liu H., Wang X. Z., Zhu S. (2014). Annexin A2 and CD105 expression in pancreatic ductal adenocarcinoma is associated with tumor recurrence and prognosis. *Asian Pacific Journal of Cancer Prevention*.

[8] Han Y., Ye J., Dong Y., Xu Z., DU Q. (2015). Expression and significance of annexin A2 in patients with gastric adenocarcinoma and the association with E-cadherin. *Experimental and Therapeutic Medicine*.

[9] Yee D. S., Narula N., Ramzy I. (2007). Reduced annexin II protein expression in high-grade prostatic intraepithelial neoplasia and prostate cancer. *Archives of Pathology & Laboratory Medicine*.

[10] Onishi M., Ichikawa T., Kurozumi K. (2015). Annexin A2 regulates angiogenesis and invasion phenotypes of malignant glioma. *Brain Tumor Pathology*.

[11] Zhang W., Gao C., Zhang S., Fang G. (2017). Serum Annexin A2 level is associated with diagnosis and prognosis in patients with oral squamous cell carcinoma. *Journal of Oral and Maxillofacial Surgery*.

[12] Maji S., Chaudhary P., Akopova I. (2017). Exosomal Annexin II promotes angiogenesis and breast cancer metastasis. *Molecular Cancer Research*.

[13] Liu X., Ma D., Jing X., Wang B., Yang W., Qiu W. (2015). Overexpression of ANXA2 predicts adverse outcomes of patients with malignant tumors: a systematic review and meta-analysis. *Medical Oncology*.

[14] Shi H., Xiao L., Duan W. (2016). ANXA2 enhances the progression of hepatocellular carcinoma via remodeling the cell motility associated structures. *Micron*.

[15] Milligan M. J., Harvey E., Yu A. (2016). Global intersection of long non-coding RNAs with processed and unprocessed pseudogenes in the human genome. *Frontiers in Genetics*.

[16] Kalyana-Sundaram S., Kumar-Sinha C., Shankar S. (2012). Expressed pseudogenes in the transcriptional landscape of human cancers. *Cell*.

[17] Wu C., Wei Y., Zhu Y. (2018). Identification of cancer-related potential biomarkers based on lncRNA-pseudogene-mRNA competitive networks. *FEBS Letters*.

[18] Tutar L., Ozgur A., Tutar Y. (2018). Involvement of miRNAs and pseudogenes in cancer. *Methods in Molecular Biology*.

[19] Hu X., Yang L., Mo Y. Y. (2018). Role of pseudogenes in tumorigenesis. *Cancers*.

[20] Li S., Zou H., Shao Y. Y. (2017). Pseudogenes of annexin A2, novel prognosis biomarkers for diffuse gliomas. *Oncotarget*.

[21] Cheng A. L., Kang Y. K., Chen Z. (2009). Efficacy and safety of sorafenib in patients in the Asia-Pacific region with advanced hepatocellular carcinoma: a phase III randomised, double-blind, placebo-controlled trial. *The Lancet Oncology*.

[22] Niu L.-l., Cheng C.-l., Li M.-Y. (2018). ID1-induced p 16/IL6 axis activation contributes to the resistant of hepatocellular carcinoma cells to sorafenib. *Cell Death & Disease*.

[23] Medavaram S., Zhang Y. (2018). Emerging therapies in advanced hepatocellular carcinoma. *Experimental Hematology & Oncology*.

[24] Llovet J. M., Montal R., Sia D., Finn R. S. (2018). Molecular therapies and precision medicine for hepatocellular carcinoma. *Nature Reviews. Clinical Oncology*.

[25] Tong M., Che N., Zhou L. (2018). Efficacy of annexin A3 blockade in sensitizing hepatocellular carcinoma to sorafenib and regorafenib. *Journal of Hepatology*.

[26] Zhang L., Ge C., Zhao F. (2016). NRBP2 overexpression increases the chemosensitivity of hepatocellular carcinoma cells via Akt signaling. *Cancer Research*.

[27] Zhang H., Yao M., Wu W. (2015). Up-regulation of annexin A2 expression predicates advanced clinicopathological features and poor prognosis in hepatocellular carcinoma. *Tumour Biology*.

[28] Shaker M. K., Abdel Fattah H. I., Sabbour G. S. (2017). Annexin A2 as a biomarker for hepatocellular carcinoma in Egyptian patients. *World Journal of Hepatology*.

[29] Abdelkarim B. T. M., Maranda V., Drouin G. (2017). The fate of retrotransposed processed genes in Arabidopsis thaliana. *Gene*.

[30] Hayashi H., Arao T., Togashi Y. (2015). The *OCT4* pseudogene *POU5F1B* is amplified and promotes an aggressive phenotype in gastric cancer. *Oncogene*.

[31] Liu J., Xing Y., Xu L., Chen W., Cao W., Zhang C. (2017). Decreased expression of pseudogene PTENP1 promotes malignant behaviours and is associated with the poor survival of patients with HNSCC. *Scientific Reports*.

[32] Ma H., Ma T., Chen M., Zou Z., Zhang Z. (2018). The pseudogene-derived long non-coding RNA SFTA1P suppresses cell proliferation, migration, and invasion in gastric cancer. *Bioscience Reports*.

[33] Sun M., Nie F. Q., Zang C. (2017). The pseudogene DUXAP8 promotes non-small-cell lung cancer cell proliferation and invasion by epigenetically silencing EGR1 and RHOB. *Molecular Therapy*.

[34] Qian Y. Y., Li K., Liu Q. Y., Liu Z. S. (2017). Long non-coding RNA PTENP1 interacts with miR-193a-3p to suppress cell migration and invasion through the PTEN pathway in hepatocellular carcinoma. *Oncotarget*.

[35] De Martino M., Palma G., Azzariti A., Arra C., Fusco A., Esposito F. (2017). The *HMGA1* pseudogene 7 induces miR-483 and miR-675 upregulation by activating *Egr1* through a ceRNA mechanism. *Genes*.

[36] Chan W. L., Yuo C. Y., Yang W. K. (2013). Transcribed pseudogene *Ψ*PPM1K generates endogenous siRNA to suppress oncogenic cell growth in hepatocellular carcinoma. *Nucleic Acids Research*.

[37] Boye T. L., Jeppesen J. C., Maeda K. (2018). Annexins induce curvature on free-edge membranes displaying distinct morphologies. *Scientific Reports*.

